# Laboratory Investigation Comparing Plaque Removal Efficacy of Two Novel-Design Toothbrushes with Different Brushing Techniques

**DOI:** 10.3390/dj6020008

**Published:** 2018-04-07

**Authors:** Wansiri Jansiriwattana, Thitiwan Teparat-Burana

**Affiliations:** 1 Dental Department, Nakhon Phanom Hospital, 270, Apibaanbancha Rd., Nakhon Phanom 48000, Thailand; wansiri.jw@gmail.com; 2 Department of Oral Medicine and Periodontology, Faculty of Dentistry, Mahidol University, 6 Yothi Street, Rajthevi, Bangkok 10400, Thailand

**Keywords:** manual toothbrush, dental plaque, laboratory study, professional brushing

## Abstract

Manufacturers of manual toothbrushes have improved novel brush head designs aimed at achieving good plaque removal even with inadequate brushing technique. This study tested the plaque removal efficacy of two novel designs compared with a flat trimmed toothbrush with different brushing techniques. Two novel-design toothbrushes (Colgate^®^ 360° Surround and Oral-B^®^ Pro-Health™ Clinical Pro-Flex) were tested. The control toothbrush was Butler^®^ GUM 311. Artificial plaque was applied on artificial teeth. Brushing with the modified Bass and horizontal scrub technique was then performed independently. After brushing, the remaining plaque index and Proximal Marginal Index (PMI) were evaluated. With the same brushing technique, there was no statistically significant difference in the mean of the whole mouth plaque scores or PMI among the three different toothbrush designs with neither brushing techniques (*p* > 0.05). When a comparison was made between the mean PMI of the two brushing techniques in each toothbrush design, Colgate^®^ showed no statistically significant difference with either brushing technique (*p* > 0.05), but Butler^®^ and Oral-B^®^ showed statistically significantly less PMI with modified Bass technique than with horizontal scrub technique (*p <* 0.05). No difference in the whole mouth plaque removal efficacy was found among the three different toothbrush designs with either brushing technique.

## 1. Introduction

Periodontal diseases are complex processes between microorganisms and host responses. The accumulation of supragingival plaque results in the developmental of gingivitis [1,2]. Plaque must be prevented from accumulating on the tooth surface, or it must be removed before it produces inflamed changes in the gingiva [1]. Manual tooth brushing is widely accepted as a highly effective method for supragingival plaque removal. The amount of plaque removal accomplished by the general population is still undesirable because of brushing technique and inadequate brushing time. Posterior teeth are often less favorably cleaned than anterior teeth [3], and the proximal surfaces have been shown to have the most plaque accumulations [4].

A horizontal scrubbing motion is the most common technique used by uninstructed individuals [5]. Bass technique was first described in 1954 [6], and in the past decade has become the method most frequently recommended. This brushing method is generally accepted as an effective means for bacterial plaque removal adjacent to and directly beneath the gingival margin, at least 1 mm subgingivally [7]. Bass [6] stated that the brush should clean the proximal surfaces as far as the bristles would go by directing them at an angle of about 45°. Katz et al. [8] then recommended the modification of the Bass method combining this technique with Roll method named “Modified Bass technique” to ensure the complete plaque removal of both coronal surfaces and gingival margins. The Department of Health, Ministry of Public Health of Thailand [9] has recommended the Modified Bass technique as the proper toothbrush method for the general population. Bass described the optimal characteristics of a toothbrush as being flat trimmed bristles [10]. Nevertheless, the specifications of a standard toothbrush according to the American national standard or the American dental association (ADA) standard in 2008 [11], as well as Thai industrial standard in 2005 [12], did not include specifications of the bristle of the toothbrush that accord with Bass in 1948 [10].

Ideally, toothbrush designs should be user-friendly, with high efficacy in plaque removal, and not harmful to soft tissues or hard tissues. Manufacturers of manual toothbrushes have improved to overcome inconsistent and varied toothbrush motion by introducing novel brush head designs aimed at achieving good plaque removal even with inadequate brushing technique [13]. However, world workshops on plaque control and oral hygiene practices have consistently concluded that there is insufficient evidence to support any brush design as superior to another [14]. Saxer & Yankell [15] concluded that the ideal toothbrush should give maximum access to the proximal areas, regardless of the brushing technique used, which remains a challenge for those involved in toothbrush development. Numerous new toothbrush designs have been introduced featuring modifications to the brush heads (i.e., angled bristles away from brush head, multi-leveled bristles) in order to improve efficacy. Recently, tapered bristles with a cleaning tip and dentist-like polishing cups have been incorporated into a manual toothbrush. In addition, another novel design of manual toothbrush with two flexible pressure-sensitive wings adjusted to the contour of teeth and gums and crisscross bristles is introduced into a market. It is interesting to know whether these novel designs of toothbrush are different in terms of plaque removal efficacy when used with different brushing techniques.

The present study is a laboratory trial, designed to evaluate the plaque removal efficacy of two novel-design toothbrushes compared with a manual flat trimmed control toothbrush when the toothbrush is used with different brushing techniques: horizontal scrub vs. modified Bass.

## 2. Materials and Methods

### 2.1. Toothbrushes

**Figure 1:**
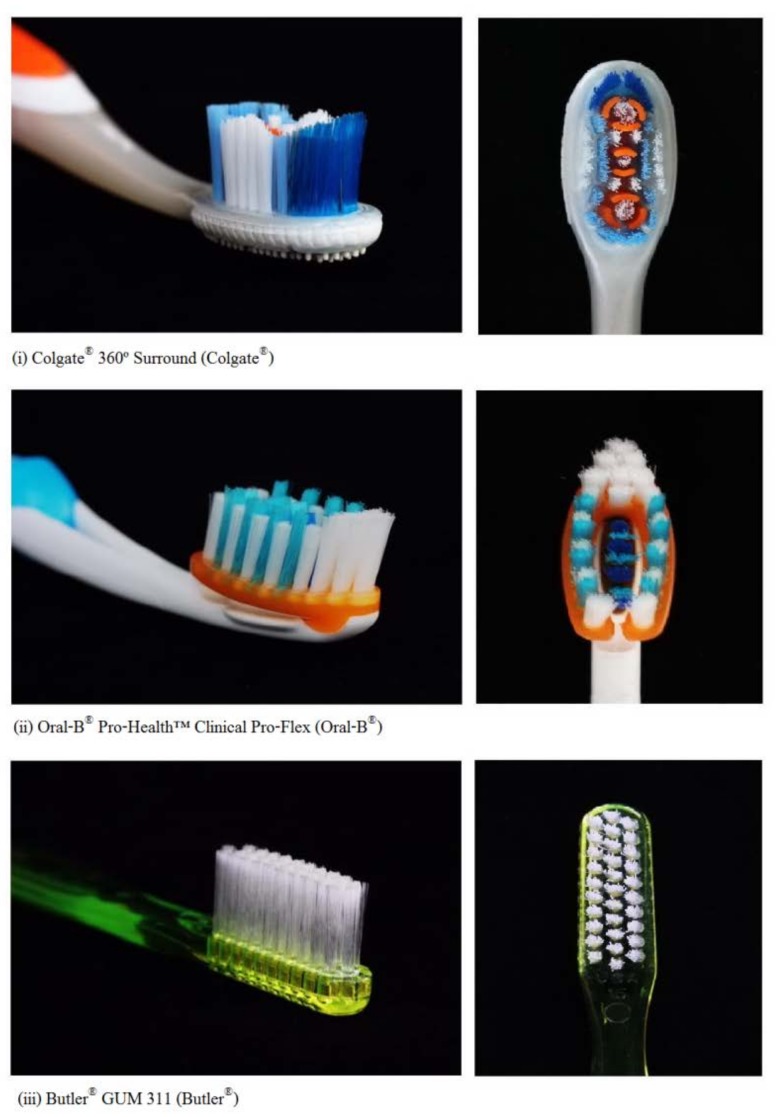
The three manual toothbrushes tested.

In this study, three manual toothbrushes were used: (i) a soft, tapered bristles, different bristle size, pointed and extended outer bristles with raised cleaning tip and the soft rubber polishing cups design: Colgate^®^ 360º Surround (Colgate-Palmolive, New York, NY, USA), (ii) a soft, end-rounded bristles, angled crisscross bristles with raised cleaning tip design: Oral-B^®^ Pro-Health™ Clinical Pro-Flex (The Procter & Gamble Co., Cincinnati, OH, USA), and (iii) a soft, multi-tufted toothbrush, end-rounded bristle, with the bristle tuft all of the same length and positioned perpendicular to the handle, in three rows: Butler^®^ GUM 311 (Sunstar Butler, Chicago, IL, USA) as manual control toothbrush (Figure 1).

### 2.2. Study Design

The study had a randomized single-use model. Randomization was performed by randomly selecting the sealed opaque envelopes which contain the type of toothbrush and brushing technique. Each toothbrush was single-used brushing for a total of 11 tests of each toothbrush design as well as each brushing technique (modified Bass or horizontal scrub technique), for a total of 66 tests. To reduce the bias of treatment results, after the brushing procedure was done, each test was labeled with a code, and then plaque assessment was performed later.

### 2.3. Brushing Procedure

**Figure 2:**
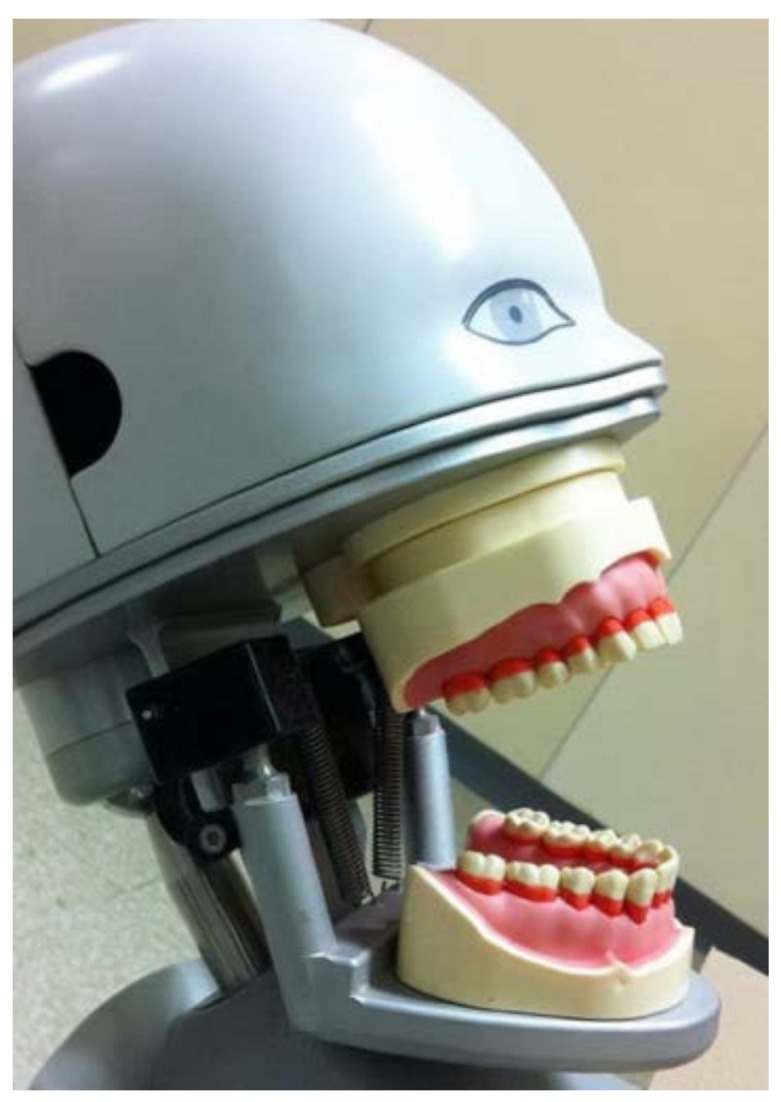
Artificial plaque on artificial teeth before brushing procedure.

Professional brushing was performed by one calibrated dentist, who provided a standardized brushing force of 250 g by using the electronic scale before every test, with the method of brushing defined by an expert. This study modified the method previously described by Danser [16]. Briefly, upper and lower dentoforms with 28 removable artificial teeth, pink soft silicone gingiva type (Nissin^®^, Kyoto, Japan) are mounted to the articulator of dental stimulator (Nissin^®^, Kyoto, Japan), which was set in the same position in all tests. Before each brushing cycle, artificial teeth were applied with denture high spot indicator (Arti-Spot^®^, Bausch Articulating Paper Inc., Nashua, NH, USA), which was used as a plaque substitute for 5 min on the test surface, on the cervical half of the clinical crown of all artificial teeth on the buccal and lingual sides [17] (Figure 2).

Toothbrushes were selected for testing in a randomized manner, and a brush head was submerged in tap water for 75 s. Then, all surfaces of the teeth were cleaned except on occlusal surface by the calibrated dentist for 2 min (1 min for upper and 1 min for lower artificial teeth, starting from buccal surface of either left or right posterior teeth to the other side and then on the lingual surface with the same manner) with a brushing force of 250 g without dentifrice. After each brushing test, the residual artificial plaque was completely removed with acetone solution and water. Then the artificial teeth were completely dried before the next brushing cycle.

### 2.4. Plaque Assessment

After a 2-min brushing cycle, artificial plaque remaining on the dentoform was calculated using the plaque index of O’Leary et al. 1972 [18], which calculates the percentage of the remaining plaque presented on all artificial teeth, in 6 individual tooth surfaces (buccal, lingual, mesio-buccal, mesio-lingual, disto-buccal, disto-lingual) on the dentogingival junction area. In addition, Proximal Marginal Index by Benson et al. 1993 [19] evaluated the remaining plaque on the gum line surfaces in the buccal and lingual surfaces of 6 index teeth: maxillary right lateral incisor, maxillary right first molar, maxillary left first premolar, mandibular left lateral incisor, mandibular left first molar and mandibular right first premolar. Each surface is divided into 3 unequal segments: disto-proximal and mesio-proximal from the line angle to the papilla and the marginal surface 3 mm upward from the margin. Then, all 3 areas can be scored and related as an average or reported separately, i.e., as proximal or marginal. Plaque in each of the segments is scored using the criteria described by Turesky et al. [20] as:

0 = no plaque
1 = separate flecks of plaque covering less than 1/3 of the area
2 = discrete areas or bands of plaque covering less than 1/3 of the area
3 = plaque covering 1/3 of the area
4 = plaque covering more than 1/3 but less than 2/3 of the area
5 = plaque covering 2/3 or more of the area

### 2.5. Statistical Analysis

Two-way analysis of variance (ANOVA) was performed on the mean of the whole mouth plaque scores to evaluate the effect of toothbrush designs and brushing techniques on plaque removal efficacy. To compare Proximal Marginal plaque index between two brushing techniques, the Mann-Whitney U test was employed. The Kruskal-Wallis test was applied to compare plaque index among the three toothbrush designs. For all the tests, values of *p <* 0.05 were accepted as statistically significant.

## 3. Results

The mean of the whole mouth plaque scores after brushing are presented in Table 1. No statistically significant difference in the mean of plaque scores was found among the three different toothbrush designs with either brushing techniques (*p* > 0.05).

The mean Proximal Marginal index data are presented in Table 2. With the same brushing technique, there was no statistically significant difference of mean plaque index on either the marginal or the proximal area among the three toothbrush designs (*p* > 0.05). There was no statistically significant difference for all of 3 toothbrush designs (*p* > 0.05) when comparing the mean plaque index on buccal and lingual surfaces between the two brushing techniques. When comparing the mean plaque index on mesial surface between two brushing techniques in each toothbrush designs was made, Colgate^®^ 360° Surround toothbrush showed no statistically significant difference with either of the brushing techniques (*p* > 0.05), but Butler^®^ GUM 311 and Oral-B^®^ Pro-Health™ Clinical Pro-Flex showed statistically significant lower plaque index with modified Bass technique than with the horizontal scrub technique (*p* < 0.05). When comparing the mean plaque index on the distal surface between the two brushing techniques for each toothbrush design was made, only Butler^®^ GUM 311 showed statistically significant improved plaque removal with modified Bass technique than with horizontal scrub technique (*p* < 0.05).

## 4. Discussion

The results from the laboratory trial demonstrated that the novel designs of toothbrush have a comparable efficacy for whole mouth plaque removal. When focusing on proximal and marginal surfaces, the Proximal Marginal Index by Benson [19] was used. It was found that all three toothbrush designs had no statistically significant difference when used with either technique. However, when Oral-B^®^ Pro-Health™ Clinical Pro-Flex and a conventional design (Butler^®^ GUM 311) were used with a modified Bass technique, they showed a better plaque removal efficacy than horizontal scrub technique on proximal areas. Colgate^®^ 360° Surround showed no statistically significant difference in plaque removal efficacy between the two techniques.

The Bass technique has become the most frequently recommended method [21]. From the results, it appears that no matter what toothbrush designs were used, modified Bass technique, which represented the instructed individuals, was more effective than horizontal scrub which is used by the majority of individuals who have never been instructed in brushing [5], especially on proximal surfaces. This finding is in agreement with previous studies. Gibson & Wade [22] showed that the Bass technique was superior to the Roll technique in cleaning the tooth surface close to the gingival margin on lingual and facial aspects. But no significant differences were shown in overall effectiveness. In another study [23], it was demonstrated that the Bass technique is more effective in plaque removal from lingual side than Roll, circular scrub and horizontal scrub. The comparative study [24] of effectiveness of modified Bass and individual’s own brushing technique in plaque removal showed that the modified Bass technique is better in buccal and lingual aspects.

The general population not only brush their teeth for a shorter than optimal time, but also with inappropriate technique during routine brushing that have limited effectiveness for plaque removal from certain tooth surfaces. The gingival margins and the proximal surfaces of premolars and molars are hard to reach for plaque control [25,26]. Because of inadequacies in brushing technique, manual toothbrush manufacturers have introduced features into their designs aimed at improving plaque removal from all tooth surfaces. This study was designed to evaluate the efficacy of plaque removal not only on buccal and lingual sites but also on proximal areas. Therefore, this study showed that the designs of toothbrush do not affect plaque removal efficacy. Brushing technique is more important.

In the present study, professional brushing was performed by the same dentist, who was trained and calibrated the force in order to have accurate and standardized brushing. This was designed in an attempt to control as many variables as possible, including duration of brushing, manual dexterity and the novelty effect, according to the several studies [27,28,29,30]. The reproducibility in brushing procedures was shown due to the coefficient of variation (CV) when the control toothbrush (Butler^®^ GUM 311) was used with the horizontal scrub technique (CV = 4.80%, data not shown) and with modified Bass technique (CV = 5.38%, data not shown). However, this model may not simulate results seen with self-brushing, the dentist might be aware of the product used, and the amount of residual plaque and muscle fatigue could also be confounding factors affecting the results. Due to calibration, the dentist focused primarily on correct brushing time and brushing technique with each test so that the awareness of residual plaque would not affect the result from the brushing procedure. Instead, it would be more reliable, and the bias would be reduced, if the brushing person and the plaque investigator were different persons. In this study, muscle fatigue was controlled by limiting the number of tests per session, not more than 4 tests, with a resting period of about 10–15 min after each brushing cycle. Regarding plaque removal efficacy related to the aspect of wearing of the toothbrush, there was a study that showed that old toothbrushes have reduced effectivity in plaque removal when compared with the new toothbrushes [30,31]. A single-use brushing model was described in previous studies [32,33]. Therefore, the present study was designed to employ a single-use brushing model in an attempt to eliminate the wearing of toothbrush bristle on plaque removal efficacy.

In this study, pre-brushing plaque substitute covered on the cervical half of the clinical crown of artificial teeth tends to mimic 48 h of plaque accumulation in the human clinical study, because on the coronal half of the clinical crown, self-cleansing from food is often able to remove the plaque; thus, we refrained from painting this area with the artificial plaque. The benefit of the red-colored plaque substitute was to provide optimal contrast between the artificial teeth and the artificial gingiva. Laboratory studies can be carried out rapidly; however, they may not necessarily give an accurate representation of the clinical situation, since individual variation exists with respect to jaw and tooth formation, age, interproximal spaces, diet, mastication, tongue action, etc. [34].

Toothbrushes alone or combined with toothpaste have been proposed to cause tooth wear and abrasion of gingival tissues [35,36,37,38]. This laboratory study is lack of evaluation of potential harm to hard and soft tissues. These novel designs of toothbrush still need to have further clinical evaluations.

## 5. Conclusions

No difference in whole mouth plaque removal efficacy was found among the three different designs of toothbrush with neither brushing techniques. The significant differences were found only on plaque removal on proximal surface when Butler^®^ GUM 311 and Oral-B^®^ Pro-Health™ Clinical Pro-Flex was used with the modified Bass technique.

## Figures and Tables

**Table 1:** Mean whole mouth plaque scores (%) (SD) after brushing.

		Toothbrush	
Technique	Butler^®^ GUM 311	Colgate^®^ 360° Surround	Oral-B^®^ Pro-Health™ Clinical Pro-Flex
Horizontal scrub	70.88 (3.40)	70.02 (3.07)	68.56 (3.29)
Modified Bass	67.64 (3.64)	67.48 (6.64)	67.80 (5.09)

Statistically significant difference, *p* < 0.05.

**Table 2:**
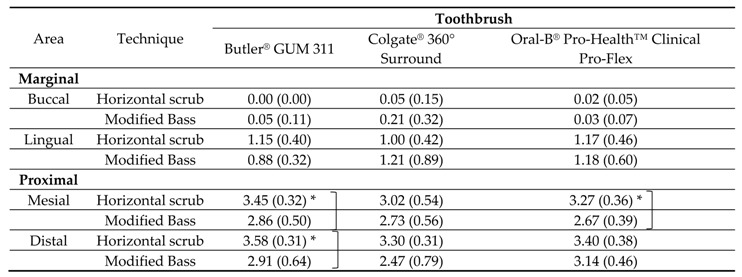
Mean Plaque Marginal index (SD) after brushing.

* Statistically significant difference, *p* < 0.05.
